# Lung Cancer Classification Employing Proposed Real Coded Genetic Algorithm Based Radial Basis Function Neural Network Classifier

**DOI:** 10.1155/2016/7493535

**Published:** 2016-11-30

**Authors:** I. Jasmine Selvakumari Jeya, S. N. Deepa

**Affiliations:** ^1^Department of Computer Science and Engineering, Hindusthan College of Engineering and Technology, Coimbatore, Tamil Nadu 641 032, India; ^2^Department of Electrical and Electronics Engineering, Anna University, Regional Campus, Coimbatore, Tamil Nadu 641 046, India

## Abstract

A proposed real coded genetic algorithm based radial basis function neural network classifier is employed to perform effective classification of healthy and cancer affected lung images. Real Coded Genetic Algorithm (RCGA) is proposed to overcome the Hamming Cliff problem encountered with the Binary Coded Genetic Algorithm (BCGA). Radial Basis Function Neural Network (RBFNN) classifier is chosen as a classifier model because of its Gaussian Kernel function and its effective learning process to avoid local and global minima problem and enable faster convergence. This paper specifically focused on tuning the weights and bias of RBFNN classifier employing the proposed RCGA. The operators used in RCGA enable the algorithm flow to compute weights and bias value so that minimum Mean Square Error (MSE) is obtained. With both the lung healthy and cancer images from Lung Image Database Consortium (LIDC) database and Real time database, it is noted that the proposed RCGA based RBFNN classifier has performed effective classification of the healthy lung tissues and that of the cancer affected lung nodules. The classification accuracy computed using the proposed approach is noted to be higher in comparison with that of the classifiers proposed earlier in the literatures.

## 1. Introduction

In the growing scenario, the potential method for improving the retention of survival of a patient is detection of the cancer at an early stage. Since the past decades, cancers such as breast cancer, cervical cancer, lung cancer, blood cancer, throat cancer, and mouth cancer have been noted to occur. The present approach focuses on various analysis and classification of lung module of the human body. Considering various lung diseases like fibrosis, carcinoma, and so on, lung cancer plays a major role in increasing the death rate in both men and women. From the current statistics it is noted that around 1.25 million people possess the lung cancer disease and almost around 1.18 million people die because of this disease [1]. The patient's survival rate can be increased on detecting cancer at an early stage. Detecting lung cancer at the beginning stage is a hectic task. In general, several patients are detected with the occurrence of lung cancer in the middle or at the advanced stage of cancer. To identify the presence of lung cancer and to detect the abnormalities in the initial stage computer aided diagnosis helps in the diagnostic process.

In this approach, lung cancer classification is performed employing the proposed RCGA based RBFNN. From the earlier approach, it is lucid that the classification is carried for lung cancer images using the developed self-regulated gray wolf optimizer based extreme learning machine classifier model. It is noted to achieve classification accuracy better than the already available classifiers but is noted to get hang over with that of the local optima problem during the convergence for solutions. As a result, this approach proposed RBFNN classifier combined with RCGA for lung cancer classification avoiding local minima problem and enabling faster convergence of the network.

Further to the above proposed RCGA based RBFNN classifier, this approach also contributes to the feature extraction part of the considered lung healthy and cancer images from LIDC and real time datasets from hospitals. This approach focuses on introducing second-order statistical features extracted using Run Length Matrices (RLM) and Gray Level Cooccurrence Matrices (GLCM), which play a major role in providing the inputs to the proposed classifier model and facilitate the proposed RCGA based RBFNN classifier to perform effectively and efficiently for lung cancer classification.

Introducing RCGA in the present approach avoids the Hamming Cliff problem encountered with that of the BCGA, and this RCGA tunes the weights and bias values of RBFNN classifier model for faster convergence with accurate classification solutions. The metrics considered for classification analysis hold the same as presented in the existing approach. Simulation proves that the proposed RCGA-RBFNN classifier model is able to achieve better classification accuracy than that of the other classifiers available in the literature and as well that of the classifier proposed in the existing approach.

## 2. Proposed Feature Extraction Approach of Lung CT Images

Feature extraction transforms the given input data represented by the medical images to be diagnosed into its relevant features. Basically, several approaches have been proposed ranging from studying the characteristics of the primitive texture elements, or textons (features), employing statistics of the individual pixel values, modeling the images with random field models, and filtering with kernels [2]. This present approach focuses on extracting the features from the input medical images employing second-order statistics, which includes, Gray Level Run Length Matrices (GLRLM) and GLCM. These second order statistics perform feature extraction based on the properties of pixel pairs and thus employs gray level run-length measures and GLCM. The RLM and GLCM features tend to attain higher discrimination indexes and these cannot be detected visually. One of the most popular second-order statistical texture methods employed for medical images seems to be the GLCM proposed by Haralick [3].

### 2.1. Gray Level Run Length Matrix: Feature Extraction Measure

GLRLM is a higher order statistical measure employed for determining features and results in quantitative values of the features for the given image [4]. Subjectively, in a specified direction, the consecutive pixels with the same gray level constitute a gray level run and the number of pixels present in the gray level run formulates the run length. In a given image, run length matrix can be computed based on the given length “*l*” of the entire image. For a particular image with “*n*” pixels, the size of the run length matrix will be considered to be *M* × *N*, where *M* and *N* represent the maximum gray level and the maximum run length possible in a particular image, respectively. From the definition, it is clear that short gray level run lengths denote fine features and long gray level run lengths occurs and denotes coarse features. Features are computed based on the relationships between the run lengths. More short run lengths result in fine texture with similar gray level intensities; on the other hand, long run lengths result in coarse texture. Numerous features are derived using the RLM for the considered images.  Table 1 presents the descriptors extracted from the RLM and the formulae employed for calculating these descriptors. In Table 1, *p*(*i*, *j*) indicates the pixel values corresponding to the images with “*i*” rows and “*j*” columns.

For the considered image, the gray level run length matrices can be computed with respect to any given direction [5]. The RLM can be computed for four principle directions, 0°, 45°, 90°, and 135°. On computing the GLRLM along each direction, these features derived can be employed either based on the direction or hybridizing all the directions to obtain a global view of the feature information on the images.

### 2.2. Gray Level Cooccurrence Matrices (GLCM): A Feature Extraction Measure

GLCM is defined as the joint probability pertaining to the occurrence of gray levels for the two pixels *i* and *j* specified on spatial relationship in an image. GLCM is also called Spatial Gray Level Dependency (SGLD) matrix. Fundamentally, the spatial relationship is defined with respect to distance “*d*” and angle “*θ*”. If the texture is fine and if the distance “*d*” is comparatively larger than that of the texture size, the gray levels of points are then separated based on distance “*d*” which will be different, in a manner that SGLD matrices spreads out relatively in a uniform manner compulsorily. On the other hand, when the texture is found to be coarse and the distance “*d*” is found to be smaller in comparison with that of the size of the texture elements, the pairs of points located at the distance “*d*” then possess similar gray levels.  Table 2 presents the statistical measure that form the GLCM and all these measures average the feature values in all the four directions.

## 3. Proposed Real Coded Genetic Algorithm for RBFNN Classifier Tuning

In the present approach, RCGA is developed and is employed to tune the necessary parameters of RBFNN, which acts as a classifier for performing the classification of lung cancer images. Basically, Genetic Algorithm (GA) is a generalized search and optimization approach which is inspired by the theory of biological evolution process [12]. Over the decades, it has been noted that GA has been applied for numerous system identification problems, control problems, data classification problems, image classification problems, and so on. It should be noted that the traditional GA possess Hamming Cliff problem which results in certain difficulties during the coding of continuous variables. Thus, the focus is on implementing RCGA to overcome the Hamming Cliff problem and to tune the parameters of RBFNN classifier. The following section will elucidate the flow of the proposed RCGA.

### 3.1. Genetic Algorithm: Revisited

GA is a stochastic evolutionary optimization algorithm employed for problem solving and for determining the solutions to optimization and search problems [13]. The GA makes the population to get evolved by looping itself over an iteration process. The algorithm operates by maintaining the population of individuals, which represents the prominent solutions in the search space. The individuals are randomly generated in the search space and their fitness is computed. They are suitably selected based on the evaluation of their fitness and allowed to pass through the generations for computing the optimal solutions. The selected individual members in the population pool are allowed to pass through the various genetic operations for computing new individual solutions with the best fitness value. The basic GA is given as follows:

- Start: randomly generate genetic populations for “*n*” chromosomes.
- Evaluation: evaluate the fitness for each chromosome generated in the population.
- New Population: new population can be created by repeating the following steps until the required solution is computed.
  1. Selection: the chromosomes with the best fitness will be allowed to pass through the selection process that is out of the randomly generated individual chromosomes; two best (parent) chromosomes (possessing best chromosome) will be selected to perform the forthcoming operations.
  2. Crossover: crossover is performed for the best two selected parents to produce offsprings. Crossover enables developing offsprings of complete new breed which tends to achieve effective solution. Crossover operates with a crossover probability. When no crossover is performed, offspring will be the exact copy of the parent chromosomes.
  3. Mutation: mutation also enables producing new offsprings based on the chromosomes generated from the crossover. Mutation operates with mutation probability. In mutation process, new offsprings are produced based on the locus points (locus indicates the position in chromosome).
- Evolution process: place newly generated offsprings in place of the existing population.
- Replacement and Looping: for the newly generated population, evaluate the fitness and proceed with the algorithmic flow.
- Termination: test for stopping condition; and return the best optimal solution computed.

**Figure 1:**
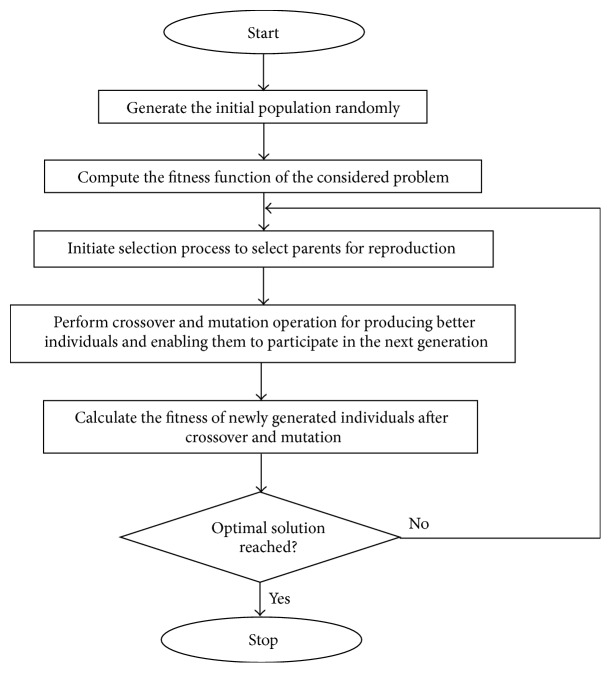
Basic flowchart of genetic algorithm process.

Figure 1 shows the basic flowchart of GA process. From Figure 1, it can be observed that the process of GA starts with initializing the population, the information gets exploited and is contained in the present population and the GA explores the solution space to form new individuals by generating children employing the genetic operators, namely, selection, crossover (recombination), and mutation. These operators possess the capability for replacing the members of the old generation. The individuals with higher probabilities are allowed to participate and flow through the next generation. As generations pass by, the algorithm converges to the best chromosome, resulting in an optimum or near optimal solution.

### 3.2. Proposed Real Coded Genetic Algorithm for RBFNN Tuning

In a basic GA, the solution variables are represented employing the binary strings. Basically, the fundamental GA locates the neighborhood search space of optimal or near optimal solutions, but the main disadvantage is that it needs larger number of generations for convergence. This requirement for a larger number of generations is due to the fact of decoding the binary strings into real numbers and the reverse encoding of real numbers to binary strings. The applicability of binary strings in basic GA makes it encounter the Hamming Cliff problem. This problem occurs when 10000 and 01111 possessing the neighboring points in the phenotype are noted to have maximum hamming distance in the genotype search space. Each and every bit has to be reversed simultaneously to overcome this Hamming Cliff problem. This reversal probability of occurrence in crossover and mutation operation is very minimal and likely to result in premature convergence. To avoid this premature convergence and Hamming Cliff problem, modifications are made in the proposed RCGA such that the bias is represented using integer numbers and weights are represented using floating point number. The supremacy of GA is based on its operators which tend to build new chromosomes and aim to determine better solutions to the problem. On viewing the literature background, few of the real coded genetic operators employed for various applications include the following:

- Rank selection, arithmetic crossover, and random mutation [14]
- Roulette wheel selection, weighted mean crossover, and uniform mutation [15]
- Roulette wheel selection, max-min arithmetical crossover, and uniform mutation [16]
- Tournament selection, BLX-a crossover, and random mutation as employed by Alcalá et al. [17]

This approach contribution employs roulette wheel selection, BLX-*μ* crossover, and nonuniform mutation for carrying out the RCGA flow avoiding the presence of premature convergence. The details of the operators employed in the proposed RCGA are presented in the following sections.

#### 3.2.1. Roulette Wheel Selection

The fundamental idea of roulette wheel selection in GA flow is that a linear search is made through a roulette wheel possessing the slots in the wheel that are weighted in proportion to that of the individual's fitness values. An individual with the best fitness as of then in the population will contribute more towards the solution space, but when this does not tend towards the solution space, the following chromosome in line has a chance, and the current chromosome becomes weak. The process of roulette wheel selection to select the better individuals to participate in the next step is as follows.

**Step 1 .**

Compute the cumulative sum of the total expected value of the individuals generated in the population. Consider it to be “*C*_*s*_”.

**Step 2 .**

Perform Steps 3 and 4 for “*N*” times, meaning a better individual is chosen, till then.

**Step 3 .**

Select a random integer “*i*” between 0 and *C*_*s*_.

**Step 4 .**

Sum the evaluated fitness values over the generation for the individuals generated in the population until the sum becomes equal to or greater than “*i*”. The selected individual is the one, whose expected fitness value makes the sum exceed the specified limit.

**Step 5 .**

Stop.

The rate of evolution in roulette wheel selection depends on the variance of fitness noted in the population.

#### 3.2.2. BLX - *μ* Crossover

Crossover is a recombination operator which produces new offsprings based on the parent chromosomes. This operator combines the chromosomes' individual best position from the randomly generated population. BLX - *μ* crossover operator determines a new position “*p*” from the extended search space [*α*_1_, *α*_2_] as given below in (1) and (2):

(1) $$\begin{array}{c} p=\left\{\begin{array}{cc} \alpha_{1}+c\times \left(\alpha_{2}-\alpha_{1}\right)\text{:} & if\;\;v_{min}\leq p\leq v_{max},\\ repeat\;\;sampling\text{:} & otherwise, \end{array}\right. \end{array}$$

where,

(2) α1=v1−μ×v2−v1,α2=v2+μ×v2−v1;

“*c*” is the uniform random number between 0 and 1.

**Figure 2:**
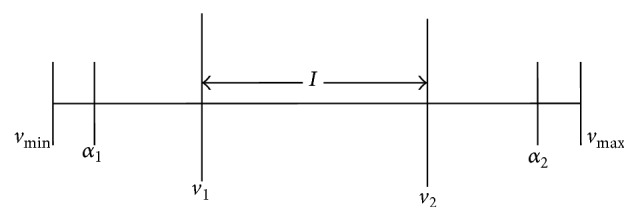
BLX - *μ* crossover.

Figure 2 shows the BLX-*μ* crossover operation with respect to one-dimensional case. It is inferred from Figure 2 that *α*_1_ and *α*_2_ will lie between *v*_min_ and *v*_max_, the variable's lower and upper bounds, respectively. On carrying out numerous trial runs, it is noted that *α* = 0.5 provides better results.

The main feature of this BLX - *μ* crossover operator is the created position point that depends on the location of both the parent chromosomes. When both parent chromosomes are close to each other, the new search point will also be close to the parents and when the parent chromosomes are very far from each other the positional search will then be more in a random manner. Once the crossover operation is completed, the fitness pertaining to the individual's best search position is compared with that of the two offsprings and the best one is taken to be the new individual best position.

#### 3.2.3. Nonuniform Mutation

In GA process, mutation is a varying operator wherein the values are randomly changed at one or more positions with respect to the selected particle. In this nonuniform mutation, for each chromosome *Y*_*i*_^*n*^ = {*y*_1_, *y*_2_, *y*_3_,…, *y*_*m*_} in the population of *n*th generation, an offspring *Y*_*i*_^*n*+1^ = {*y*_1_′, *y*_2_′, *y*_3_′,…, *y*_*m*_′} is generated as

(3) yk′=yk+Δn,upperbound−yk,if  a  random  λ  is  0,yk+Δn,yk−lowerbound,if  a  random  λ  is  1,

where upper bound and lower bound are the lower and upper bounds of the variables *y*_*k*_. The function Δ(*n*, *y*) returns a value in the range [0, *y*] such that Δ(*n*, *y*) approaches zero as *n* increases. This enables to uniformly search the space in the initial stages (when *n* is small), and very locally at the later stages. This nonuniform mutation strategy increases the probability of the generation of a new number close to its successor, rather than being a random choice. The search function Δ(*n*, *y*) is evaluated as

(4) $$\begin{array}{c} \Delta \left(\;n,y\;\right)=y\cdot \left(\;1-\beta^{\left(1-n/N\right)q}\;\right), \end{array}$$

where *β* is a random number from [0,1], *N* is the maximum iteration, *q* is a system parameter which determines the degree of dependency on the number of generations.

Thus, the proposed RCGA employs these three operators for tuning the weights and bias of the RBFNN classifier.

## 4. Proposed RCGA Based Radial Basis Function Neural Network Classifier for Lung Cancer Classification

Over the decades, numerous statistical and machine learning procedures were devised to perform medical image classification and, amidst all the approaches, first and second generation Artificial Neural Networks (ANNs) were widely employed. In these algorithms, Support Vector Machine (SVM) was one of the most important algorithms and is applied for medical image classification problems [18]. Numerous literatures have reported the applicability of SVM in replacement for conventional neural networks reducing the computational complexity and the time involved during the training process [9]. In this manner, extreme learning machine classifier was found to provide good solutions for complex tasks and thus modification ELM classifier was proposed [10]. Due to the local minima problem encountered by the proposed ELM classifier, this approach aims to develop RBFNN classifier tuned by proposed RCGA to carry out lung cancer classification.

### 4.1. Generalized Radial Basis Function Neural Network Classifier

Fundamentally, RBFNN classifier models are employed for approximation, classification, and prediction applications due to their learning capability of not getting hanged over with global or local minima or maxima, which seems to occur more frequently in several other neural network classifiers like multilayer perceptron, adaline, Back Propagation Neural Network (BPNN), Hopfield neural network, and so on. With respect to commonly employed neural classifiers, RBFNN classifier is noted to achieve faster convergence with minimal training time.

**Figure 3:**
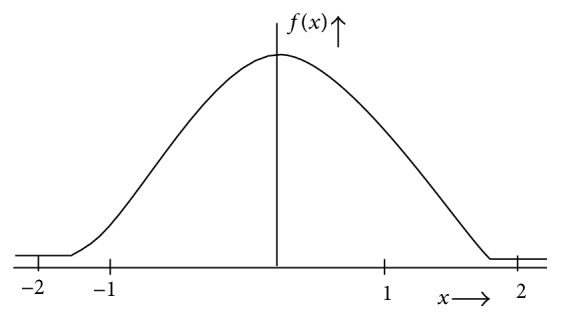
Nonlinear Gaussian Kernel function.

RBFNN classifier employs a nonlinear activation function called as Gaussian function, which is tuned by adjusting the spread value or represented with that of the kernel functions. This nonlinear kernel activation function is less prone to the problem related to the nonstationary input because of the behavior of RBFNN hidden units. Gaussian kernel function possesses a curve with peak at zero distance and this function tends to decrease as the distance from the centre starts increasing. Gaussian nonlinear activation function is defined by

(5) $$\begin{array}{c} f\left(\;x\;\right)=e^{-2x}, \end{array}$$

where “*x*” represents the net input of RBFNN model.  Figure 3 represents the radial basis Gaussian kernel function employed for the proposed work.

The parametric design of RBFNN classifier is the computation of spread and weight of output mode and its structural design is the architectural enhancement of the number of neurons involved. The output of RBFNN is computed employing

(6) $$\begin{array}{c} Y=\overset{n}{\underset{k=1}{\sum }}f\left(\;\left\|\;X-C_{k}\;\right\|\;\right)\times W_{jk},\;for\;\;j=1,\ldots ,n, \end{array}$$

where “*X*” means the input vector, “*C*_*k*_” is the *k*th centre node in the hidden layer, *W*_*jk*_ refers to the weights between the hidden and output layer, and “*f*” represents the nonlinear Gaussian kernel function.

### 4.2. Proposed RBFNN Classifier Algorithm Tuned with Real Coded Genetic Algorithm

**Figure 4:**
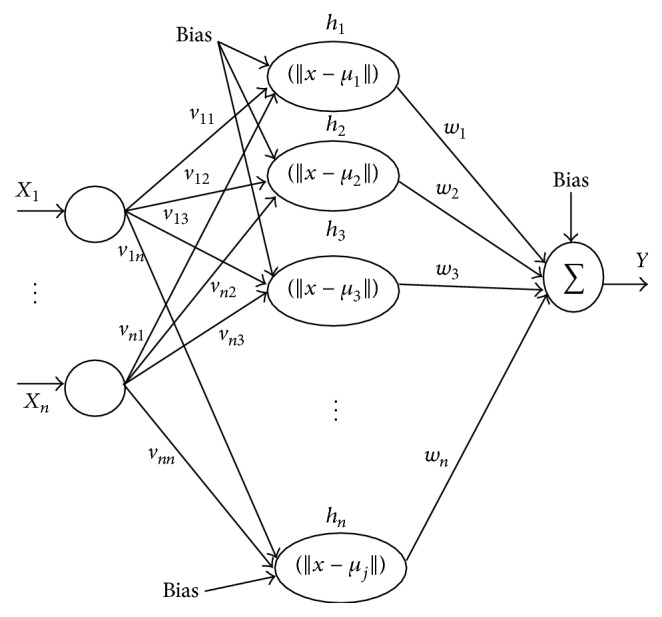
Proposed RBFNN classifier model.

The contribution in the present approach involves the development of a modified radial basis neural network classifier with its parameters tuned using RCGA to achieve better learning convergence, classification solutions, and minimal error rates. In general, neural networks spatial information is not considered for the conventional radial basis function and only identical distribution of data are considered. The related spatial autocorrelation is incorporated into the modeled RBFNN classifier to be implemented for lung cancer classification.  Figure 4 shows the architecture of the proposed RBFNN classifier model.

Basically, the contextual and structural data are not obtained after the segmentation and classification process. On performing spectral classification, the class to which it belongs is only inferred. To achieve better recognition and classification with respect to the intrinsic properties like attributes size, shape, and length and semantic knowledge; it is required to incorporate spatial information into the model. This information can be incorporated into the model employing Gaussian processes. The *Y* output unit has *W*_*ok*_ as bias and *Z* hidden unit has *V*_*ok*_ as bias.

#### 4.2.1. Proposed Algorithm for RCGA Tuned RBFNN Classifier

The proposed algorithm for carrying out effective lung cancer classification employing RCGA tuned RBFNN classifier is as follows.

**Step 1 .**

Initialize the weight between the input layer to hidden layer and between hidden layers to output layer to small random values. Initialize the parameters for RCGA process.

**Step 2 .**

Initialize the momentum factor and learning rate parameter.

**Step 3 .**

When the stopping condition is false do Steps 4–11.

**Step 4 .**

For each the training dataset pair do Steps 5–10.

**Step 5 .**

Each input unit belonging to the input layer receives the input signals *x*_*i*_ and transmits this signal to all the units in the hidden layer above, namely, to the hidden units.

**Step 6 .**

Each hidden layer unit (*z*_*j*_, *j* = 1,…, *p*) sums the received weighted input signals as

(7) $$\begin{array}{c} z_{-inj}=v_{oj}+\overset{n}{\underset{i=1}{\sum }}x_{i}v_{ij}, \end{array}$$

applying the continuous Gaussian activation function at this point as

(8) $$\begin{array}{c} Z_{j}=f\left(\;z_{inj}\;\right),\;\text{i.e},f\left(Z_{inj}\right)=e^{-Z_{inj2}}, \end{array}$$

and sends this signal to all the units in the layer above, namely, output units.

**Step 7 .**

For each of the output unit (*y*_*k*_, *k* = 1,…, *m*), compute its net input as

(9) $$\begin{array}{c} y_{-inj}=w_{ok}+\overset{p}{\underset{j=1}{\sum }}z_{j}w_{jk}. \end{array}$$

**Step 8 .**

Apply Gaussian activation function to the net input to calculate the output signals as

(10) $$\begin{array}{c} Y_{k}=f\left(\;y_{-ink}\;\right),\;\text{i.e},\;f\left(Y_{inj}\right)=e^{-Y_{inj2}}. \end{array}$$

**Step 9 .**

Each output unit (*y*_*k*_, *k* = 1,…, *m*) receives a target pattern corresponding to an input pattern; error information term is calculated as

(11) $$\begin{array}{c} \delta_{k}=\left(\;t_{k}-y_{k}\;\right)f^{\prime }\left(\;y_{-ink}\;\right). \end{array}$$

**Step 10 .**

Each hidden unit (*z*_*j*_, *j* = 1,…, *n*) sums its delta inputs from the units in the layer above as

(12) $$\begin{array}{c} \delta_{-inj}=\overset{m}{\underset{k=1}{\sum }}\delta_{j}w_{jk}. \end{array}$$

Error information term is calculated as

(13) $$\begin{array}{c} \delta_{j}=\delta_{-inj}f^{\prime }\left(\;z_{-inj}\;\right). \end{array}$$

**Step 11 .**

Compute the weight correction term between the output unit and the hidden unit given by

(14) $$\begin{array}{c} \Delta w_{jk}=\alpha \delta_{k}z_{j}+\mu \Delta w_{jk}\left(\;\text{old}\;\right). \end{array}$$

And the bias correction term is given by

(15) $$\begin{array}{c} \Delta w_{ok}=\alpha \delta_{k}+\mu \Delta w_{ok}\left(\;\text{old}\;\right). \end{array}$$

**Step 12 .**

Compute the weight correction term between the hidden unit and the input unit given by

(16) $$\begin{array}{c} \Delta v_{ij}=\alpha \delta_{j}x_{i}+\mu \Delta v_{ij}\left(\;\text{old}\;\right). \end{array}$$

And the bias correction term is given by

(17) $$\begin{array}{c} \Delta v_{oj}=\alpha \delta_{j}+\mu \Delta v_{ok}\left(\;\text{old}\;\right). \end{array}$$

**Step 13 .**

Each output unit (*y*_*k*_, *k* = 1,…, *m*) updates its bias and weights (*j* = 0,…, *p*) given by

(18) wjknew=wjkold+Δwjk,woknew=wokold+Δwok.

**Step 14 .**

Each hidden unit (*z*_*j*_, *j* = 1,…, *p*) updates its bias and weights (*i* = 0,…, *n*) given by

(19) vijnew=vijold+Δvij,vojnew=vojold+Δvoj.

**Step 15 .**

Check the error values in (11) and (13). If the minimum set error value is reached, go to Step 24; else, proceed to the following step.

**Step 16 .**

Invoke proposed RCGA with its necessary initialized parameters.

**Step 17 .**

Evaluate the fitness (error) values as per (11) and (13) for the current population. The current population will be the current weight and bias values.

**Step 18 .**

If best fitness is not reached, do Step 19; else, go to Step 24.

**Step 19 .**

Perform roulette wheel selection.

**Step 20 .**

Perform BLX - *μ* crossover.

**Step 21 .**

Perform nonuniform mutation.

**Step 22 .**

Evaluate fitness for the current offspring (weights and bias) generated.

**Step 23 .**

If necessary condition is met, go to Step 24; else, go to Step 6.

**Step 24 .**

Test for the stopping condition of the RBFNN model.

The stopping condition can be the number of iterations reached; minimize MSE value until the learning rate gets decreased to a particular value.

Hence, the proposed RCGA with RBFNN classifier determines the optimal weight values between the input and hidden layers and as well between the hidden and output layers and bias so that the fitness (MSE) reaches the minimum value for achieving better generalization performance considering the advantages of employing Gaussian Kernels in RBFNN classifier and RCGA. The proposed RCGA based RBFNN classifier combines the features of RCGA into RBFNN classifier for computing the optimal weights and bias for making the MSE minimal.

## 5. Experimental Results and Discussion

In this paper, proposed RCGA based Radial Basis Function Nearest Neighbor Classifier is applied for lung cancer classification and the computed results are presented in the following subsections. The proposed algorithm is simulated in MATLAB R2012a environment for the medical image datasets and executed in a PC with Intel core i5 processor with 3.5 GHz speed and 2 GB RAM with 64 bit operating system.

**Figure 5:**
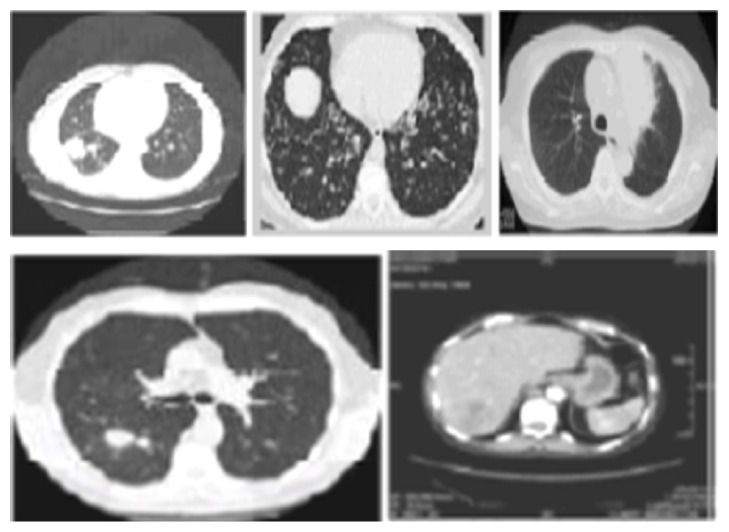
Other sample lung cancer images.

As presented in the previous approach, there exist primary and secondary stage lung cancer nodules with four various kinds of nodules, namely, well-circumscribed nodules, vascularized nodules, juxtapleural nodules, and pleural-tail nodules.  Figure 5 presents few other lung cancer images employed in the proposed algorithm. The parameter metrics for analysis of the proposed algorithm are considered to be the same as those in the previous approach, which includes sensitivity, specificity, classification accuracy, and area under Receiver Operating Characteristics (ROC).

### 5.1. Proposed Feature Analysis Process and Segmentation of Lung CT Images

The proposed feature analysis method is applied for the lung image samples collected from the LIDC database and as well for the lung image real time datasets collected from the surrounding hospitals.

As presented earlier, Haralick et al. suggested 14 statistic measurements to describe GLCM created from a moving window [11]. However, it should be noted that few of these measures are highly correlated and only a few are recommended to be employed for lung cancer classification due to the fact they are more suitable for describing features in natural scenes [19]. Henceforth, the present approach focuses on six commonly used GLCM measurements: Contrast, Entropy, Angular Second Moment, Homogeneity, Variance, and Correlation as given in Table 2. Contrast is a measure of the local variation present in the image. It increases for high contrast Region of Interest (ROI). Homogeneity or the inverse difference moment refers to the image homogeneity such that a smooth image gives a high value. This increases for low contrast ROI due to their dependence on (*i* − *j*)^2^. Entropy is a factor on the disorder of the image.

The highest value for entropy is reached when all the probabilities are equal. For smooth ROI, entropy takes low values. The angular second moment is also called energy and is a measure of the regularity in pixel patterns and homogenous images possessing high values. Variance is a general measure on heterogeneity and is strongly correlated for first-order statistical variable such as standard deviation. On the considered image being completely uniform, its variance is 0. Variance is noted to increase when the gray level values differ from their mean. Correlation is a gray level linear dependence between the pixels at the specified positions relative to each other.

With respect to the features extracted using RLM, after the calculation of RLM numerous feature descriptors are computed to capture the feature properties and differentiate among the different features. Out of the eleven descriptors of RLM, only few are recommended for lung cancer classification due to the fact that they provide a global view of the required feature information needed to classify the lung images. Hence, the present approach focuses on six commonly used RLM descriptors, namely, Short Run Emphasis (SRE), Long Run Emphasis (LRE), Short Run Low Gray level Run Emphasis (SRLGE), Short Run High Gray level Run Emphasis (SRHGE), Long Run Low Gray level Run Emphasis (LRLGE), and Long Run High Gray level Run Emphasis (LRHGE). The features are extracted as per the six descriptors of RLM and the GLCM matrices, respectively, and their average values are as tabulated in Table 3.

**Figure 6:**
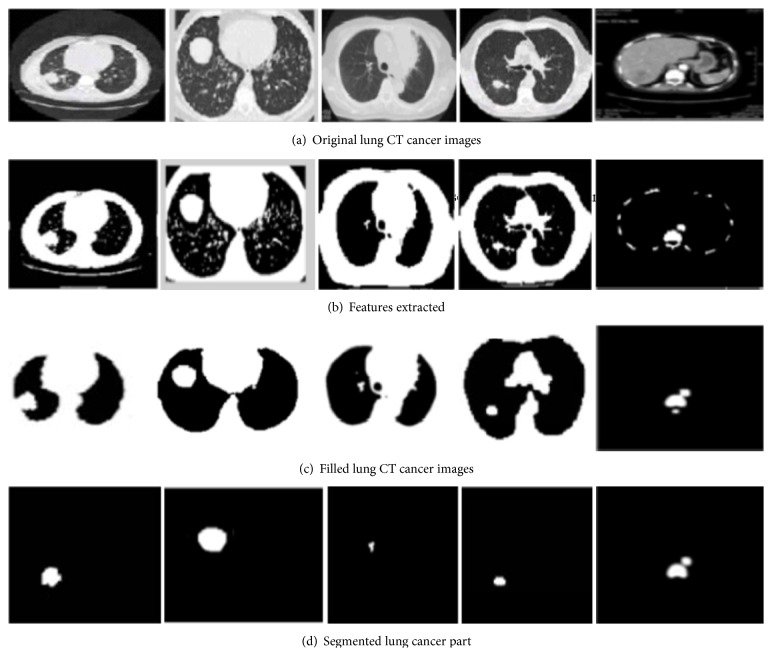
Proposed feature extraction and segmentation of lung cancer images.

The morphological operations are carried out for segmenting the lung images. Initially, the gray scale image is converted into binary image. The input image pixels with an intensity greater than that of a threshold level are replaced with a value “1” and the remaining pixels with the intensity less than that of the threshold level are replaced with a value “0”. The morphological operation is carried out to the binary image and the regularly employed structuring element based morphological operation is performed. The main advantage of having morphological operation is its speed and its simplicity.  Figure 6 shows the lung cancer image with the final segmented cancer part.

### 5.2. Lung Cancer Classification with Proposed RCGA Based RBFNN Classifier

The proposed RCGA based RBFNN classifier is now invoked for the segmented output for efficient and accurate cancer classification. All the details on the simulation process and the performance of the proposed RCGA-RBFNN classifier for the considered datasets are presented in this section. The weights and bias between the input layer and the hidden layer and between the hidden and output layer of the Radial Basis Function neural architecture are optimized employing the proposed RCGA. The inputs to the RBFNN classifier are 12 and the hidden neurons (*H*) is determined as 3 with the help of the best parameters selected with RCGA. The number of outputs specifies each of the lung nodules, namely, well-circumscribed nodules, vascularized nodules, juxtapleural nodules, pleural-tail nodules, and healthy lung without cancer tissues. Consequently, the structure of the feed forward RBFNN is 12-3-5.  Table 4 presents the algorithmic parameters for the proposed RCGA based RBFNN classifier model.

Table 5 compares the result of the proposed RCGA-RBFNN classifier simulation with the existing tested classifiers and that of the proposed SRGWO-ELM classifier in the previous approach. The RCGA-RBFNN classifier classification performance is noted to be appreciably good. Hence, it can be noted that the proposed RBFNN classifier outperforms the standard binary linear SVM, BPNN, and ELM Classifier for medical image classification and proves better classification of healthy and lung cancer nodules. Further, it is noted that there exists a significant difference in the computation time among the considered approaches.

**Figure 7:**
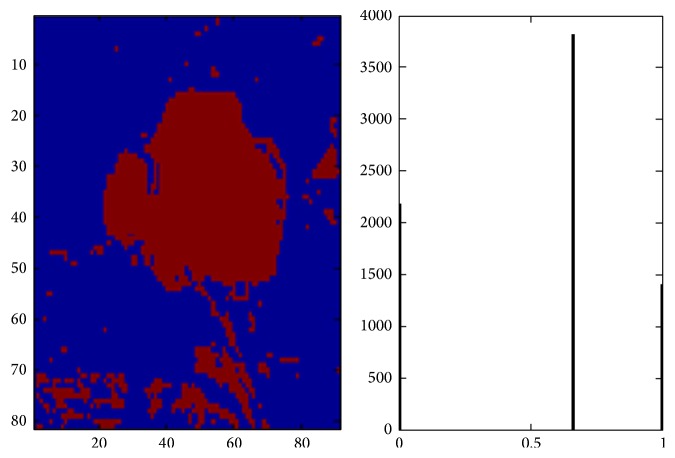
Lung cancer classification using the proposed RCGA-RBFNN algorithm.

The proposed RCGA-RBFNN classifier showed the highest overlap with the manual segmentation carried out for all the tissues.  Table 6 shows the average value based on the cancer segmentation and classification methods based on feature extraction model and compared to the state of art techniques [6–8]. The proposed RCGA-RBFNN model proves better and resulted in improved values on sensitivity, specificity, classification accuracy, and area under Receiver Operating Characteristics (AUROC) for the lung cancer datasets. The algorithm segmented lung cancer part with its histogram is as shown in Figure 7.

From Table 6, it can be inferred that the proposed RCGA-RBFNN classifier approach is noted to achieve the highest classification accuracy for both the cancer datasets from the database consortium as well as the datasets from the hospitals and diagnostic centre. Also, it can be noted that the area under the curve value is 1 for LIDC database and tends towards 1, proving the reliability nature of the proposed algorithm.

### 5.3. Lung Cancer Classification Analysis Using Proposed RCGA-RBFNN Classifier

The aim of the proposed work is to correctly classify healthy and cancer images. In the analyses of the images, each image is classified into one of two classes (either healthy or cancer affected images). Based on the features that have been extracted employing RLM and GLCM, the proposed RCGA-RBFNN classifier separates the same. Combining the publically available dataset (LIDC with healthy lung and with cancer affected) and as well the real time datasets (from Accura diagnostic centre and PSGIMSR, Coimbatore), datasets of 152 and 141 patients are analyzed, respectively. Based on the extracted 6 RLM features and 6 GLCM features, the lung cancer nodule segments are obtianed. These segments are noted to well seperate the two classes, that is, affected and not affected. The patients were selected for the respective analysis based on the average feature value of the RLM and GLCM measures.

Table 7 presents the classification accuracy computed between various classifiers with that of the proposed RCGA-RBFNN classification model. The considered images were then classified by the proposed RCGA-RBFNN classifier with leave-one-out cross-validation; that is, the classifier was trained with 29 patients and then the one patient not used in training was classified. This process is rotated in a manner such that all the patients are used as a test set.

In Table 7, it is noted that the samples chosen are 152 cases of LIDC data and 141 cases for real time data. The samples are converted into their respective pixel values and are input to the RBFNN classifier model. The considered samples were loaded in a sequential manner; and henceforth the time delay process was well within the permissible extent.  Table 8 presents few other metrics observed during the run time of the proposed RCGA-RBFNN classifier algorithm along with the existing classifiers for LIDC dataset from the literature and the SRGWO-ELM classifier proposed earlier in the approach.

**Figure 8:**
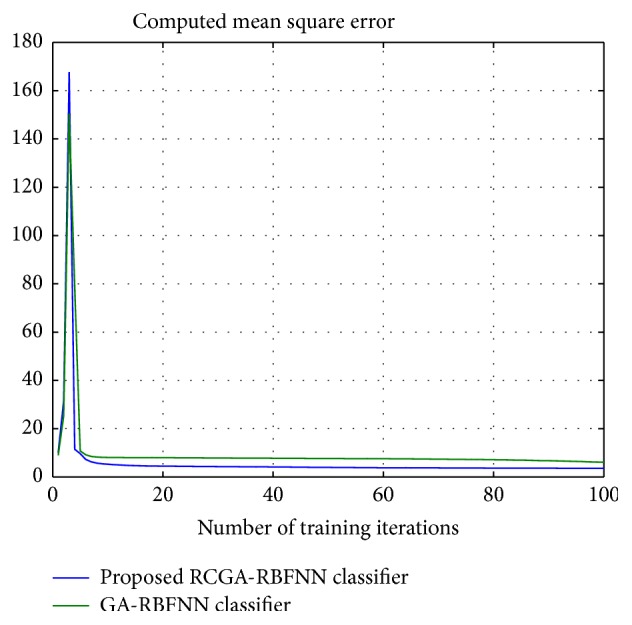
Computed MSE employing the proposed RCGA-RBFNN classifier.

**Figure 9:**
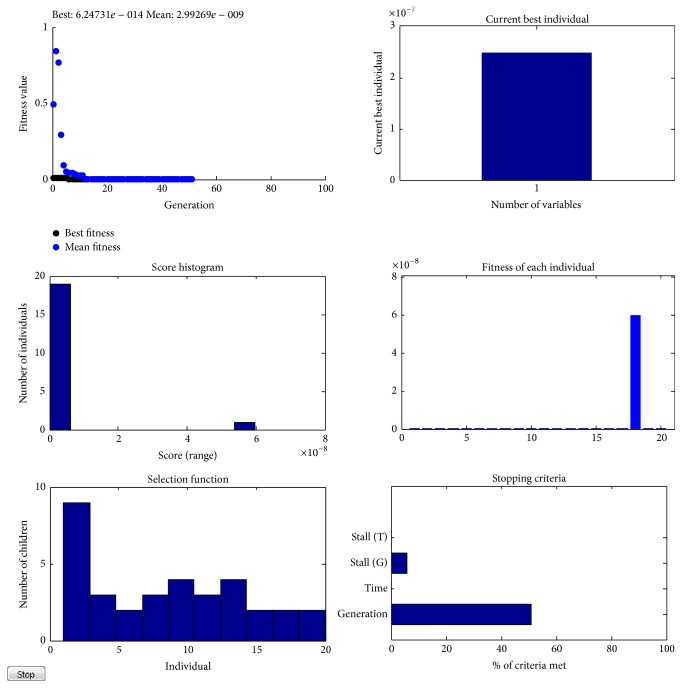
Proposed RCGA tuning process.

From Table 8, it can be inferred that the proposed classifier model is noted to achieve the best solution for the various other network training parameters considered.  Figure 8 shows the plot of the computed MSE of the regular GA-RBFNN and proposed RCGA-RBFNN approach for LIDC dataset with respect to the number of iterations. It can be noted from the plot as well that minimum MSE is obtained for the proposed RCGA-RBFNN classifier model.  Figure 9 shows the RCGA tuning process for achieving minimum MSE.

## 6. Conclusion and Future Work

RCGA based RBFNN classifier is employed to perform effective classification of healthy and cancer affected lung images. RCGA is proposed in the present to overcome the Hamming Cliff problem encountered with the BCGA. RBFNN classifier is chosen as a classifier model because of its Gaussian kernel function and its effective learning process to avoid local and global minima problem and enable faster convergence. We specifically focused on tuning the weights and bias of RBFNN classifier employing the proposed RCGA. The operators used in RCGA enable the algorithm flow to compute weights and bias value so that minimum MSE is obtained.

## Figures and Tables

**Table 1:** Features derived from RLM and their evaluation formulae.

Features derived from the run-length matrices	Evaluation formula
Short Run Emphasis (SRE)	$\frac{1}{n_{r}}\overset{M}{\underset{i=1}{\sum }}\;\overset{N}{\underset{j=1}{\sum }}\frac{p\left(i,j\right)}{j^{2}}$
Long Run Emphasis (LRE)	$\frac{1}{n_{r}}\overset{M}{\underset{i=1}{\sum }}\;\overset{N}{\underset{j=1}{\sum }}p\left(i,j\right)*j^{2}$
Low Gray-level Run Emphasis (LGRE)	$\frac{1}{n_{r}}\overset{M}{\underset{i=1}{\sum }}\;\overset{N}{\underset{j=1}{\sum }}\frac{p\left(i,j\right)}{i^{2}}$
High Gray-level Run Emphasis (HGRE)	$\frac{1}{n_{r}}\overset{M}{\underset{i=1}{\sum }}\;\overset{N}{\underset{j=1}{\sum }}p\left(i,j\right)*i^{2}$
Short Run Low Gray-level Run Emphasis (SRLGE)	$\frac{1}{n_{r}}\overset{M}{\underset{i=1}{\sum }}\;\overset{N}{\underset{j=1}{\sum }}\frac{p\left(i,j\right)}{i^{2}*j^{2}}$
Short Run High Gray-level Run Emphasis (SRHGE)	$\frac{1}{n_{r}}\overset{M}{\underset{i=1}{\sum }}\;\overset{N}{\underset{j=1}{\sum }}\frac{p\left(i,j\right)*i^{2}}{j^{2}}$
Long Run Low Gray-level Run Emphasis (LRLGE)	$\frac{1}{n_{r}}\overset{M}{\underset{i=1}{\sum }}\;\overset{N}{\underset{j=1}{\sum }}\frac{p\left(i,j\right)*j^{2}}{i^{2}}$
Long Run High Gray-level Run Emphasis (LRHGE)	$\frac{1}{n_{r}}\overset{M}{\underset{i=1}{\sum }}\;\overset{N}{\underset{j=1}{\sum }}p\left(i,j\right)*i^{2}*j^{2}$
Gray-Level Non-Uniformity (GLNU)	$\frac{1}{n_{r}}\overset{M}{\underset{i=1}{\sum }}{\left(\sum_{j=1}^{N}p\left(i,j\right)\right)}^{2}$
Run - Level Non-Uniformity (RLNU)	$\frac{1}{n_{r}}\overset{N}{\underset{j=1}{\sum }}{\left(\sum_{i=1}^{M}p\left(i,j\right)\right)}^{2}$
Run Percentage Coefficient (RPC)	$\frac{n_{r}}{p\left(i,j\right)*j}$

**Table 2:** Statistical measure of GLCM and their evaluation formulae.

Features present in GLCM	Evaluation formula
Gradient Vector	$G\left(i\right)=\sqrt{G_{x}^{2}\left(i\right)+G_{y}^{2}\left(i\right)+G_{z}^{2}\left(i\right)}$
Gradient Angles	*a*(*x*, *y*, *z*) = cos⁡^−1^⁡(*g*(*x*) · *g*(*y*) · *g*(*z*))
Euclidean Distance	$d\left(x,y\right)=\;\overset{MN}{\underset{k=1}{\sum }}\sqrt{x^{k}-y^{k}}$
Entropy	$\;\overset{M}{\underset{i}{\sum }}\;\overset{N}{\underset{j}{\sum }}P\left[i,j\right]\log P[i,j]$
Homogeneity	$\;\overset{M}{\underset{i=1}{\sum }}\;\overset{N}{\underset{j=1}{\sum }}\frac{P\left[i,j\right]}{1+\left|i-j\right|}$
Contrast	$\;\overset{M}{\underset{i}{\sum }}\;\overset{N}{\underset{j}{\sum }}{\left(i-j\right)}^{2}P\left[i,j\right]$
Energy	$\;\overset{M}{\underset{i}{\sum }}\;\overset{N}{\underset{j}{\sum }}P^{2}\left[i,j\right]$
Correlation coefficient	$\;\overset{M}{\underset{i=1}{\sum }}\;\overset{N}{\underset{j=1}{\sum }}\frac{\left(i-\mu \right)\left(j-\mu \right)P\left[i,j\right]}{\sigma^{2}}$
Variance	$\;\frac{1}{2}\overset{M}{\underset{i=1}{\sum }}\;\overset{N}{\underset{j=1}{\sum }}\left({\left(i-\mu \right)}^{2}P\left[i,j\right]+{\left(j-\mu \right)}^{2}P\left[i,j\right]\right)$
Maximum Probability	$\max_{i,j}^{M,N}\;P\left[i,j\right]$
Inverse Difference Moment	$\;\overset{M}{\underset{i=1}{\sum }}\;\overset{N}{\underset{j=1}{\sum }}\frac{P\left[i,j\right]}{{\left|i-j\right|}^{k}},\;i\neq j$
Cluster Tendency	$\;\overset{M}{\underset{i}{\sum }}\;\overset{N}{\underset{j}{\sum }}{\left(i-j-2\mu \right)}^{k}P\left[i,j\right]$

**Table 3:** Average value of the feature descriptors for the medical image datasets considered.

Feature descriptors	LIDC cancer datasets	Real time lung cancer datasets (PSGIMSR)
Average Short Run Emphasis (SRE)	1.1237	1.8936
Average Long Run Emphasis (LRE)	3.5671	4.6751
Average Short Run Low Gray-level Run Emphasis (SRLGE)	1.0546	1.7659
Average Short Run High Gray-level Run Emphasis (SRHGE)	0.9987	1.0054
Average Long Run Low Gray-level Run Emphasis (LRLGE)	4.2320	6.5783
Average Long Run High Gray-level Run Emphasis (LRHGE).	5.6342	7.0089
Average Contrast	0.0266	0.0657
Average Entropy	0.1765	0.4487
Average Angular Second Moment	0.9287	0.9651
Average Homogeneity	0.9734	0.9868
Average Variance	0.6571	0.6900
Average Correlation	0.3421	0.5764

**Table 4:** Algorithmic parameters of the proposed RCGA-RBFNN classifier.

Parameters	Proposed RCGA	Parameters	Proposed RBFNN classifier
Population size	30	Learning Rate	1
Number of generations	100	Number of Iterations	100
Crossover probability	0.1	Spread	3
Mutation probability	0.01	Momentum Factor	0.2

30 independent trial runs are taken for RCGA flow process			

**Table 5:** Results of the comparison of lung cancer nodule classification with the state of art techniques.

Image datasets	BPN	Linear SVM	GA-ELM	GWO- ELM	GA- RBFNN classifier	Proposed SRGWO-ELM	Proposed RCGA- RBFNN classifier
LIDC cancer datasets	0.841	0.900	0.935	0.951	0.941	0.975	0.983
Real time lung cancer datasets (PSGIMSR)	0.875	0.893	0.907	0.928	0.932	0.963	0.989

**Table 6:** Comparison of various lung cancer classification methods.

Image datasets	Metrics for image classification	Hybrid Logistic Regression-Artificial Neural Network Approach [6]	Hopfield Neural Network & Fuzzy Clustering Approach [7]	Back Propagation Neural Network Approach [8]	SRGWO-ELM Approach	Proposed RCGA-RBFNN Classifier Approach
LIDC cancer datasets	Sensitivity	68.75	70.37	92.12	98.77	**99.10**
	Specificity	96.45	97.18	98.67	99.63	**100.00**
	Classification accuracy	88.12	89.07	91.11	96.72	**98.36**
	AUROC	0.9050	0.9010	0.9000	0.9800	**1.00**

Real time lung cancer datasets (PSGIMSR)	Sensitivity	72.39	81.37	90.65	96.57	**98.42**
	Specificity	85.47	89.01	95.47	98.21	**99.73**
	Classification accuracy	84.69	87.65	90.65	95.69	**97.31**
	AUROC	0.7231	0.8721	0.8700	0.9758	**0.9865**

**Table 7:** Accuracy results amongst various classifiers on combining the healthy and cancer affected lung images.

Datasets (Combined for Classification)	Image Types	BPN	SVM	ELM	GWO – ELM	SRGWO – ELM	GA – RBFNN	Propose RCGA– RBFNN Classifier
Lung Image Database Consortium (LIDC) (152 cases, 48 healthy and 104 cancer affected)	Healthy	87.8%	90.6%	81.2%	93.2 %	96.1%	94.5%	98.3%
	Well circumscribed nodules	84.7%	86.1%	80.4%	92.8%	94.9%	94.1%	96.9%
	Vascularized nodules	80.8%	85.1%	79.4%	91.6%	95.3%	96.9%	98.0%
	Juxtapleural nodules	84.9%	88.3%	75.4%	89.8%	91.2%	90.6%	95.3%
	Pleural-tail nodules	78.2%	75.4%	70.8%	86.5%	89.2%	88.0%	91.6%

Accura Diagnostics Centre & PSGIMSR (141 cases, 90 healthy and 51 cancer affected)	Healthy	90.6%	94.5%	87.9%	96.5%	98.7%	98.0%	99.1%
	Well circumscribed nodules	86.4%	90.8%	84.2%	93.7%	97.4%	96.8%	98.8%
	Vascularized nodules	91.8%	93.2%	89.0%	95.1%	98.0%	95.7%	99.6%
	Juxtapleural nodules	91.6%	92.4%	88.0%	94.9%	96.2%	96.0%	98.7%
	Pleural-tail nodules	86.4%	89.7%	81.6%	94.3%	97.8%	95.9%	98.9%

**Table 8:** Comparison of the network training parameters for the LIDC dataset.

Parametric measures	Hybrid Logistic Regression-Artificial Neural Network Approach [9]	Hopfield Neural Network & Fuzzy Clustering Approach [10]	Back Propagation Neural Network Approach [11]	SRGWO-ELM Approach	Proposed RCGA-RBFNN Approach
Norm	378.67	231.98	196.72	121.47	109.54
MSE Error	0.9231	0.9745	0.1120	0.0097	0.0089
Training Efficiency % Mean	86.25	81.26	94.57	98.83	99.25
Training Efficiency % STD	6.71	6.47	5.64	6.05	5.96
Testing Efficiency % Mean	90.35	88.61	95.84	97.12	98.96
Testing Efficiency % STD	6.13	6.09	5.45	6.03	6.01
Hidden neurons	19	7	12	5	3
Accuracy %	92.8	89.07	97.14	98.64	99.03
Time min	3.27	2.96	3.5	2.01	1.76
